# Evaluating the radiosensitivity of the oral microbiome to predict radiation-induced mucositis in head and neck cancer patients: A prospective trial

**DOI:** 10.1016/j.ctro.2025.100915

**Published:** 2025-01-08

**Authors:** Andreas R. Thomsen, Elsa Beatriz Monroy Ordonez, Michael Henke, Benedikt Luka, Jörg Sahlmann, Henning Schäfer, Vivek Verma, Nadine Schlueter, Anca-Ligia Grosu, Tanja Sprave

**Affiliations:** a Department of Radiation Oncology, University Hospital of Freiburg, Robert-Koch-Strasse 3, 79106 Freiburg, Germany; b German Cancer Consortium (DKTK) Partner Site Freiburg, German Cancer Research Center, Germany; c Faculty of Medicine, University of Freiburg, Freiburg, Germany; d Hannover Medical School (MHH), Department of Conservative Dentistry, Periodontology and Preventive Dentistry, Hannover, Germany; e Institute for Medical Biometry and Statistics, Medical Center - University of Freiburg, Faculty of Medicine, University of Freiburg, Freiburg, Germany; f Department of Radiation Oncology, The University of Texas MD Anderson Cancer Center, Houston, TX, USA

**Keywords:** Head and neck cancer, Radiation therapy, Mucositis, Prediction, Keratinocytes, Oral mucosa biopsy

## Abstract

**Background:**

Predicting the occurrence and/or severity of oral mucositis (OM) before commencing radiotherapy (RT) remains very difficult. The aim of this prospective trial was to investigate whether the *ex-vivo* radiation sensitivity of oral keratinocytes from head and neck (H&N) cancer patients correlates with severe OM.

**Methods:**

Oral microbiopsies of healthy gingival mucosa were collected from 63H&N cancer patients undergoing (chemo)RT, of which 58 samples were useable. Keratinocytes from these microbiopsies underwent *ex-vivo* proliferation, irradiation, and subsequently the cell spreading assay. Tubes with the cell suspension were placed within the irradiation chamber of a ^137^Cs Gammacell 40 Exactor (Best Theratronics, Canada) and exposed to 0, 2, 4, 6, or 8 Gy at a dose rate of 0.63 Gy min^−1^. Cell suspension was then immediately pipetted into custom-made polydimethylsiloxane (PDMS) rings.

The effect of demographic and clinical parameters on the cell spreading assay were also analyzed. Systematic clinical recording of OM was conducted twice a week by a specially trained examiner.

**Results:**

Most patients had node-positive disease and cancer of the oropharynx or oral cavity. The vast majority of patients received adjuvant RT and concurrent chemotherapy. Overall, 34 (58.6 %) participants developed grade 3 OM after a median dose of 32 Gy. No patient experienced a grade ≥ 4 event. There was a correlation between the cell spreading assay area and grade 3 OM (p < 0.05), equivalent to approximately 0.5 Gy dose. Demographic and clinical parameters had no significant impact on the cell spreading assay (p > 0.05 for all).

**Conclusions:**

It is necessary to establish reliable predictors of severe OM before treatment in H&N cancer to allow early management of treatment-related sequelae. This prospective trial illustrates that the intrinsic *ex-vivo* radiosensitivity of oral keratinocytes could be correlated with RT-induced OM in patients with H&N cancer. This novel predictor requires validation in larger prospective cohorts.

## Highlights

• It is necessary to establish reliable predictors of severe oral mucositis before treatment in H&N cancer to allow early management of treatment-related sequelae.
• This prospective trial illustrates that the intrinsic *ex-vivo* radiosensitivity of oral keratinocytes could be correlated with RT-induced OM in patients with H&N cancer.
• This novel predictor requires validation in larger prospective cohorts.

## Introduction

Oral mucositis (OM) is defined as erythema, epithelial lysis, or soreness (grade 1–2) and/or extensive ulceration (grade ≥ 3). It is the most common radiotherapy (RT)-related acute toxicity in head and neck cancer (H&N) patients, since acute OM occurs in almost all patients to some degree [1], [2]. As such, this high symptom burden can cause a profound deterioration in quality of life [3], [4], and any resulting treatment interruptions can compromise the efficacy of treatment [5] by means of prolonging the overall treatment time [6], [7], [8]. Moreover, chemotherapy-induced mucositis occurs in approximately 40 % of cases (oral manifestation in 20 %), and advanced (grade ≥ 3) mucositis is a significant cause of chemotherapy dose reduction [9].

Owing to both the incidence and impact of OM, accurate prediction thereof is critical. Clinical parameters have been investigated [10], along with RT-related factors [11]. Laboratory data such as the peripheral blood neutrophil:lymphocyte ratio has also been proposed, with a high ratio being associated with severe OM in H&N cancer patients undergoing RT [12], [13]. Individual genetic sensitivity to RT has also been studied; genome-wide association studies with RT-induced toxicity endpoints have identified a number of genomic risk loci, which correlate with individual radiosensitivity [14], [15]. Whereby a discrimination between radioresistant and radiosensitive tumor types can been identified by γH2AX-nfoci assay [16].

However, an emerging yet under-studied factor potentially associated with OM is the oral microbiome; this notion has arisen from compelling data linking the gut microbiome to various systemic diseases [1], [17], [18], [19], [20]. With regard to irradiation for H&N cancer, we sought to assess whether the intrinsic radiosensitivity of the oral microbiome could predict OM in these patients. Therefore, we conducted a prospective trial to evaluate whether the *ex-vivo* radiosensitivity of oral keratinocytes (from microbiopsies of pre-irradiated oral mucosa) could be used to predict OM in this population.

## Materials and methods

This prospective study was conducted at the University Hospital of Freiburg and included H&N cancer patients undergoing primary definitive or adjuvant chemoradiotherapy (CRT) between 2017 and 2022. All procedures were approved by the Ethics Committee of the University of Freiburg (vote ETK-FR 449/16, amended by vote ETK-FR 413/17). Written informed consent was obtained in all patients. All personal data and biopsy samples were de-identified.

102 patients were recruited for the collection of healthy gingival mucosa prior to the start of (C)RT, 18 of whom were screening failures or dropouts. A further 21 participants did not receive a biopsy for logistic/medical reasons or refusal. Therefore, oral microbiopsy from healthy mucosa was performed on 63 participants. A detailed description of the microbiopsy technique has been published elsewhere [21]. A total of 58 samples were analyzable and included in this study. The remaining samples were unusable due to contamination or insufficient keratinocyte growth.

This study established the *in vitro* growth of oral keratinocytes and prospectively evaluated the prediction of OM. The visualisation of the radiation-induced impairment of cell function, such as reduced migration and proliferation, was reflected by a spreading assay procedure; in other words, greater keratinocytic radiosensitivity would result in less spreading for a given dose, which would be manifested as a greater reduction in the cell spreading assay area.

**Fig. 1:**
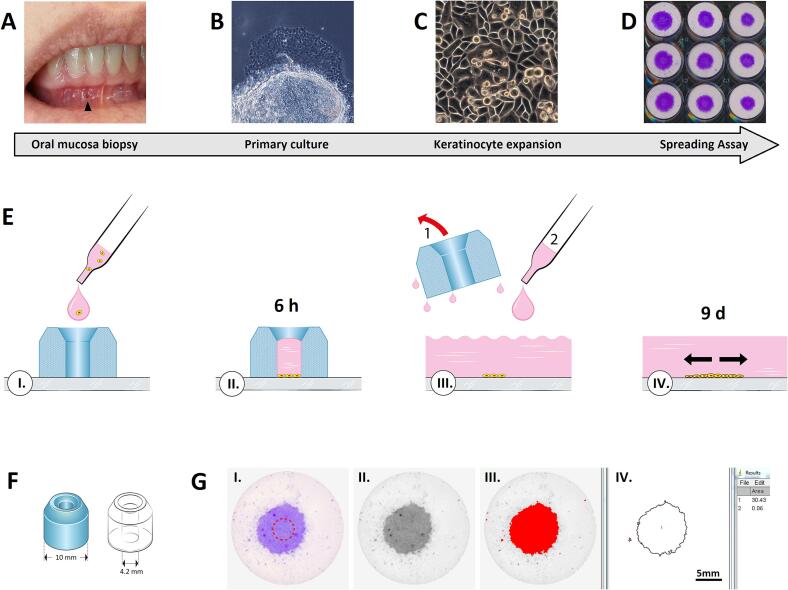
Ex-vivo radiosensitivity testing of oral keratinocytes from HN cancer patients. (A) Prior to onset of RT, a microbiopsy (arrowhead) is taken from non-attached oral mucosa of dental region 3.2 to 4.2. (B) Ex-vivo growth of oral keratinocytes is established by explant culture (C) Primary oral keratinocytes are culture-expanded. (D) Radiation sensitivity of oral keratinocytes is tested using the spreading assay. (E) Setup of the spreading assay: I. Keratinocyte suspension is pipetted into PDMS ring. II. Keratinocytes are allowed to sediment and attach. III. After 6 h, ring is removed and well is flooded with culture medium. IV. Keratinocyte cluster is allowed to spread on the culture surface for 9 days. Thereafter, clusters are fixated, stained and scanned. (F) Properties of PDMS rings: Rings are designed to attach to culture plate surface by cohesion, thereby confining initial size of keratinocyte cluster to an area of 13.9 mm^2^. (G) Measurement of cell cluster area: I. The optical scan of one keratinocyte cluster. The dashed circle illustrates the starting size of the cluster. II. Image is converted into a greyscale image. III. Application of threshold shows cell-covered area. IV. Size of keratinocyte cluster [mm^2^] is measured by image analysis. (Figures E-G modified from [22].

First, mucosal biopsies were prepared properly, and cultured *ex-vivo*. *Ex-vivo* proliferation of oral keratinocytes from the unaffected mucosa were subjected to irradiation, and subsequently the spreading assay [21]. The containers with the cell suspension were placed within the irradiation chamber of a ^137^Cs Gammacell 40 Exactor (Best Theratronics, Canada) and exposed to 0, 2, 4, 6, or 8 Gy at a dose rate of 0.63 Gy min^−1^. The scans of the cell-covered areas were then obtained from the underside of the plates using a high-resolution flatbed scanner (CanoScan 9000F Mark II, Canon Inc.) in reflected light mode and saved as TIF files. The area was quantified using the image analysis software ImageJ (NIH). The planimetric measurements mentioned above were then validated by quantifying the amount of bonded crystal violets. The violet dye was dissolved in 500 µl of 70 % ethanol per well and the optical density was measured at 590 nm in a microplate spectrophotometer. The assay procedure is demonstrated in Fig. 1 modified according to Thomsen et al. [21], [22].

All patients were discussed in a multidisciplinary tumor board. All H&N cancers were confirmed by biopsy. Before a decision on multimodality treatment was made, staging by MRI and/or CT of the thorax was performed to exclude distant metastases. Systemic therapy was administered according to current guidelines and recommendations of the tumor board. In brief, definitive (chemo)radiotherapy (CRT) was recommended for locally advanced and non-resectable tumors and was also determined by age. Adjuvant cases were eligible for CRT based on surgical pathological findings. Patients were staged according to TNM/AJCC 8th edition. All local recurrences were confirmed histologically. Demographic and treatment characteristics were extracted from the electronic patient records. Individuals with a smoking history of at least 10 pack-years were considered as smokers.

The standard dose for definitive CRT was 70 Gy EQD2 to the primary tumor region, whereas patients undergoing adjuvant RT received 60–66 Gy EQD2 to the tumor cavity. All RT was performed using intensity-modulated radiotherapy (IMRT) or helical IMRT such as tomotherapy. CT-based (Brilliance, CT Big Bore, Philips, Cleveland, OH, USA) three-dimensional treatment planning was performed (Oncentra MasterPlan, Nucletron, Veenendaal, The Netherlands; and Eclipse™ planning systems, Varian Medical Systems, Palo Alto, CA, USA), using individually collimated portals (6 or 18 MV; Synergy; Elekta, Crawley, United Kingdom), IMRT, or volumetric modulated arc therapy (VMAT). Image-guided RT was performed for all patients; since 2019, RT was performed using Surface Guided RT (C-RAD, Catalyst, C-RAD AB, Uppsala, Sweden). If required, concomitant with RT, cisplatin 40 mg/m^2^ was administered weekly. Prior to (C)RT, all patients received a dental examination and, if necessary, focal treatment or extraction. During (C)RT, all patients performed standardized daily oral care.

The main focus was a systematic recording of mucositis. For this purpose, a clinical examination of the patients was performed twice a week by a specially trained examiner. This involved systematic examination of the nine regions of the oral mucosa: hard and soft palate, left and right side, floor of the mouth, upper and lower lip, as well as left and right buccal regions. World Health Organization scores for OM were used. The severe mucositis grade 3 according to the WHO classification was defined as follows: when extensive oral ulcerations occur and oral intake consists totally of liquid food.

All patients were monitored by a surgeon and radiation oncologist every three to six months for the first two years, followed by annual visits thereafter. Acute side effects (until 90 days) were evaluated according to the Common Terminology Criteria for Adverse Events version 5.0 (CTCAE v.5). Late toxicity was judged using the modified Late Effects in Normal Tissues criteria (subjective, objective, management, and analytic, LENT-SOMA).

### Statistical analysis

The raw data of the cell spreading assay area was standardized to the area at 0 Gy and plotted. Because the standardized cell spreading assay area and radiation doses (2 Gy, 4 Gy, 6 Gy, 8 Gy) were linearly related, a linear model was created to determine the effects of radiation dose (s.a.) and the presence of grade 3 mucositis (clinically assessed in participants) on the cell-covered area (spreading assay). The linear regression was performed with mucositis as a categorical variable and radiation dose (s.a.) as a continuous variable. In addition, the effect of demographic and clinical parameters of the participants on the cell spreading assay area were analyzed. For this purpose, the cell spreading assay area was defined as the dependent variable and gender, age (<65 vs. ≥ 65 years), T-stage, N-stage, UICC stage, HPV status, smoking status, alcohol status, ECOG performance status were defined as covariates. Descriptive statistics are reported as a mean, median (range) and frequencies. P-values < 0.05 were considered statistically significant. Statistics were performed with SPSS version 29 (IBM, Armonk, NY, USA) and the open-source statistical software environment R (Version 4.4.0, R Foundation for Statistical Computing, Vienna, Austria).

## Results

A total of 58 patients treated with primary or adjuvant radio(chemo)therapy for locally advanced H&N cancer were included. The main characteristics of the study population are summarized in Table 1. Primary tumors were situated in the oropharynx (n = 28, 48.3 %), hypopharynx (n = 12, 20.7 %), and the oral cavity (n = 14, 24.1 %) (Table 1). Vast majority were HPV positive patients. Seventeen (29.3 %), 21 (36.2 %), and 12 (20.7 %) patients had T2, T3, and T4 disease, respectively. All but six (10.3 %) patients were node-positive. Extracapsular nodal spread was found in seven (12.1 %) participants.

**Table 1:** Main characteristics of patients and tumor. Patient, tumor, and therapy details characteristics of head and neck cancer patients treated by (C)RT in our institution between 2017 and 2022 (n = 58). Staging of primary head and neck cancer was based on the 8th Edition of the UICC TNM classification.

	**n (%)**
***All (n = 58)***	
**Age** (median, range)	62,4 (38–84)
**Sex**	
female	12 (20,7)
male	46 (79,3)
**Smoking**	
no	8 (13,8)
yes	50 (86,2)
**Localisation**	
nasopharynx	1 (1,7)
oropharynx	28 (48,3)
hypopharynx	12 (20,7)
oral cavity	14 (24,1)
CUP	3 (5,2)
**Histology**	
squamous cell carcinoma	57 (98,3)
mucoepithelialcarcinoma	1 (1,7)
**Grading**	
1	10 (17,2)
2	25 (43,1)
3	23 (39,7)
**T-stage**	
1	2 (3,4)
2	17 (29,3)
3	21 (36,2)
4	12 (20,7)
4a	2 (3,4)
4b	1 (1,7)
X	3 (5,2)
**N-stage**	
0	6 (10,3)
1	17 (29,3)
2	5 (8,6)
2a	2 (3,4)
2b	9 (15,5)
2c	11 (19)
3	2 (3,4)
3b	6 (10,3)
**UICC-stage**	
I	10 (17)
II	10 (17)
III	6 (10,3)
IVA	23 (39,7)
IVB	9 (15,5)
**ECS**	
no	51 (87,9)
yes	7 (12,1)
**Radiotherapy**	
adjuvante	16 (27,6)
definitive	42 (72,4)
**Concomitant chemotherapy**	
yes	50 (86,2)
**Mucositis ≥ 3**	
yes	34(58,6)
no	24(41,4)
**Alcohol**	
yes	47 (81)
no	11 (19)
**HPV status**	
yes	25 (43,1)
no	31 (53,4)
unknown	2 (3,4)
**ECOG performance status** at the baseline	
0	10 (17,2)
1	45 (77,6)
2	3 (5,1)

Abbreviation: *ECOG:* Eastern Cooperative Oncology Group performance status; *ECS:* extracapsular spread in lymph node; *HPV*: human papillomavirus, *N-stage*: lymph node involvement, *T-stage*: tumor size; *UICC TNM*: tumor staging system according to Union for International Cancer Control.

RT was administered adjuvantly in 42 (72.4 %) and definitively in 16 (27.6 %) participants. CRT was delivered to 50 (86.2 %) patients. Most patients were smokers (86.2 %) and consumed alcohol regularly (81.0 %). No wound healing disorders were observed after the biopsy of the healthy gingival mucosa before the start of therapy. During treatment, 34 (58.6 %) participants developed grade 3 OM after a median dose of 32 (range 8–69.3 Gy) Gy. No patient experienced a grade ≥ 4 event.

**Fig. 2:**
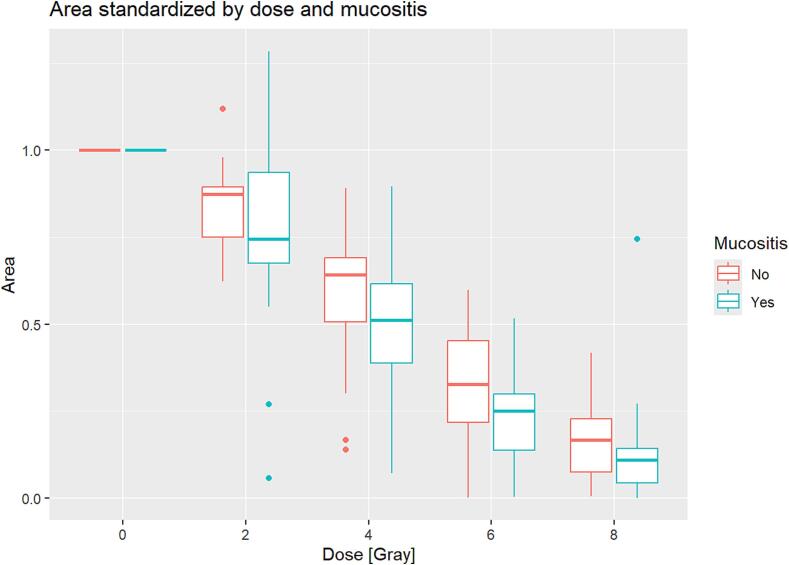
A linear model with boxplot representation of the relationship between the irradiation dose (x-axis) and the spreading assay area (y-axis). The groups were stratified according to the presence of severe grade 3 oral mucositis (yes/no).

Fig. 2 shows the association with OM and the cell spreading assay area. When comparing patients with grade 3 OM to non-severe mucositis (grade ≤ 2 OM) patients, the former had a significant impact (p < 0.05), equivalent to approximately 0.5 Gy of irradiation dose, resulting in a significantly enhanced volume decrease in the cell spreading assay area after any radiation dose (2 Gy, 4 Gy, 6 Gy, 8 Gy) compared to the non-mucositis group (p < 0.05).

When comparing the non-severe −mucositis patients to non-irradiated samples (0 Gy), a significant reduction in the cell spreading assay area was found after all irradiation doses (2 Gy, 4 Gy, 6 Gy, 8 Gy) (p < 0.05 for all) (Fig. 2). The keratinocytes and their replicates displayed inter-individual variation of the spreading assay area with a wide range (Table 2). This corresponded to a different size of the spreading assay area in the non-irradiated controls (0 Gy). Thus, the standardized areas at the respective dose level before irradiation were as follows for 2 Gy: range 0.057––1.285; 4 Gy: 0.071–0.896; 6 Gy: 0.001–0.599; 8 Gy: 0.001–0.745 (Table 2).

**Table 2:** Absolute planimetric (cm^2^) and standardized values to 0 Gy from the cell spreading assay areas replicates for the respective dose levels before irradiation.

*Absolute values (cm^2^) for cell spreading assay area before irradiation*		
**Dose**	**Min**	**Max**
0 Gy	25.9	366.3
2 Gy	1.5	297.8
4 Gy	3.1	235.4
6 Gy	0.1	150.1
8 Gy	0.1	68.3
*Standardized values for cell spreading assay area before irradiation*		
**Dose**	**Min**	**Max**
0 Gy	1	1
2 Gy	0.057	1.285
4 Gy	0.071	0.896
6 Gy	0.001	0.599
8 Gy	0.001	0.745

**Fig. 3:**
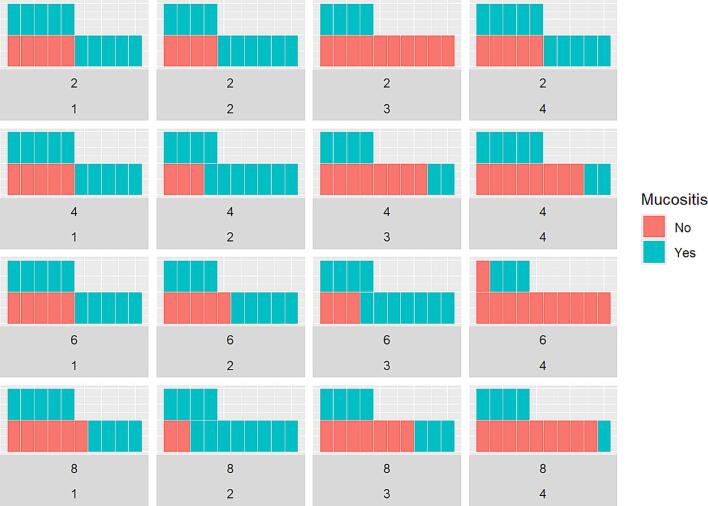
The waffle plot shows the distribution of OM grade 3 in the quartiles of the spreading assay area at 0 Gy.

Therefore, we then investigated how the initial size of the spreading assay area correlated with the development of grade 3 OM. For this purpose, the values of the cell-covered area in the unirradiated controls (0 Gy) were divided into four quartiles (Q1-Q4) and additionally stratified by presence of grade 3 OM. However, the corresponding waffle plot showed that distribution of OM grade 3 was independent of the quartiles of the spreading assay area at 0 Gy (Fig. 3).

**Fig. 4:**
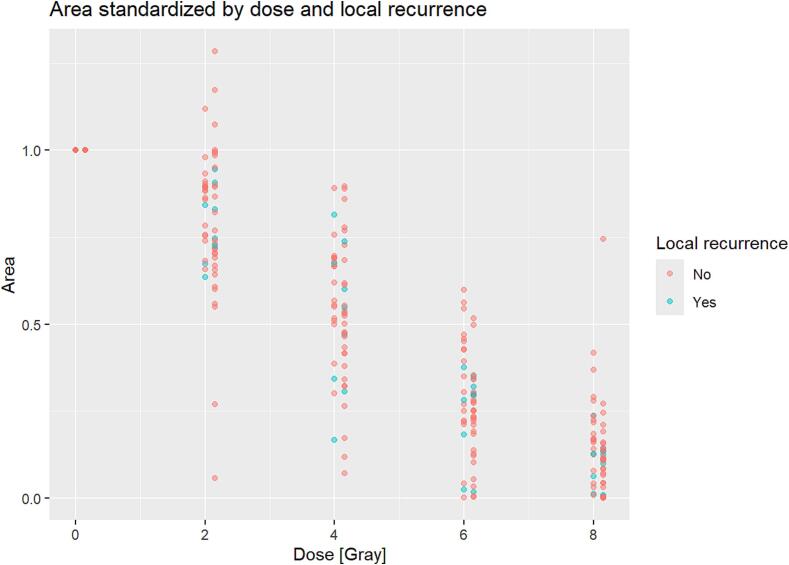
A linear model between correlation of grade 3 oral mucositis and local recurrence. The groups were stratified according to the presence of severe grade 3 mucositis (yes/no). Increasing irradiation levels are shown on the x-axis and standardised spreading assay areas are shown on the y-axis.

The demographic and clinical parameters of the participants such as gender, age (<65 vs. ≥ 65 years), T-stage, N- stage, UICC stage, HPV status, smoking status, alcohol status, and ECOG performance status had no significant impact on the cell spreading assay area (p > 0.05 for all) (Supplement Fig. 1A). Furthermore, no significant correlation was found between the local recurrence rate and the occurrence of grade 3 OM (Fig. 4).

## Discussion

It is necessary to establish reliable predictors of severe OM before treatment in H&N patients to allow early management of treatment-related sequelae. This is the first known prospective study to investigate the intrinsic radiosensitivity in H&N patients using the minimally invasive method of biopsying oral keratinocytes. It was observed that the occurrence of severe OM is significantly associated with the cell spreading assay area (p < 0.05) and therefore could predict radiotoxicity.

Mucosal keratinocytes are an essential counterpart of commensal bacteria in humans, and consequently form a basis of the microbiome model [23], which is why further analyses of the interactions between the bacteria from the saliva samples and the spreading assay from the respective patients are planned in the future. In this context, Kwak et al. confirmed the existence of the interaction between specific oral bacterial complexes and the development of H&N cancer [24]. RT-induced changes in the oral microbiota have been well-identified, along with the predictive capability for OM [19], [25]. Zhu et al. studied the oral microbiome before and during (C)RT in patients with nasopharyngeal carcinoma [19]. The authors found that the oral microbiome in case of I-II grade OM could predict the development of severe OM [19]. Consistent with these findings, Reyes-Gibby et al. showed that changes in the population density of genera under treatment were associated with the occurrence of severe OM [26]. Moreover, dynamic changes in the microbiological profile have an impact on the trajectory of OM [27]. The principle predictive value of such models based on the dynamic change of the oral microbiome is contrasted to the interventional studies with prophylactic probiotic use. The prospective randomized study by Peng et al. showed that the use of probiotics with *Streptococcus salivarius* under RT significantly reduced the severity, duration, and time to onset of OM [28].

In our study, patients with grade 3 OM had a significant effect (p < 0.05) compared to patients with non-severe mucositis (grade ≤ 2 OM), which corresponded to an additional radiation dose of about 0.5 Gy. This result was reflected in a significantly greater decrease in volume in the cell spreading test area after each radiation dose (2 Gy, 4 Gy, 6 Gy, 8 Gy) compared to the group with non-severe mucositis (p < 0.05). This allows the intrinsic radiosensitivity of the mucosa to be quantified for the first time. In other words, the intrinsic mucosal radiosensitivity causes a greater decrease in the spreading assay area, which would have corresponded to the additionally applied dose of 0.5 Gy. These results strengthen the role of intrinsic radiosensitivity in the development of severe OM, which needs to be further investigated.

The keratinocytes and their replicates displayed inter-individual variation of the spreading assay area with a wide range (Table 2). The question is whether the initial size of the spreading area correlated with the development of grade 3 OM. Surprisingly, the distribution of OM grade 3 was independent of initial spread area (Fig. 3).

Our study found no significant influence of participants' demographic and clinical parameters such as gender, age (<65 vs. ≥ 65 years), T-stage, N-stage, UICC stage, HPV status, smoking status, alcohol status and ECOG performance status on cell spread area (p > 0.05 for all). Concordant with our results, the study by Hansen et al. showed that smoking was rather protective against the development of OM [29]. In our study, no mucositis G4 was observed, despite > 50 % of locally advanced tumors. In our view, the following factors were also responsible: consistent use of IMRT, supportive inpatient care and pausing RT until clinical improvement.

Some limitations of this study should be mentioned. Firstly, this study aimed to evaluate and establish the predictive method in a small collective. Therefore, the sample may be too small to detect further statistical correlations. In addition, we did not include in our model the established extrinsic factors for the development of severe mucositis, such as the average dose to the exposed mucosa and cisplatin addition. This may lead to a bias in the correlation between *ex vivo* and clinical results.

Another major limitation of our study is the absence of validation of our results. Furthermore, the representativeness of our monocentric collective and the generalizability of the findings should be validated in large prospective studies.

## Author contributions

A.R.T. and M.H.: Study conception and study design. A.R.T., B.M., M.H., B.L., J.S., T.S: Data acquisition, data analysis and data interpretation. J.S., T.S.: Statistical analysis. A.R.T., B.M., M.H., J.S., V.V., T.S: Manuscript editing. H.S., V.V., N.S., A.L.G., and T.S.: Manuscript reviewed.

## Ethics approval and consent to participate

The study was approved by the institutional ethical review committee (reference no. ETK-FR 449/16, amended by vote ETK-FR 413/17).

## Consent for publication

Not applicable.

## Availability of data and materials

The data used in this analysis are available with the authors’ permission.

## Funding

This project was funded by Bundesministerium für Bildung und Forschung (BMBF, Germany) within project “Zielstrukturen der individuellen Strahlenempfindlichkeit (ZISStrans) (02NUK047B)”.

## Declaration of competing interest

The authors declare that they have no known competing financial interests or personal relationships that could have appeared to influence the work reported in this paper.
