# Supplementary figures and images for: Evaluating the radiosensitivity of the oral microbiome to predict radiation-induced mucositis in head and neck cancer patients: A prospective trial
Source: Clin Transl Radiat Oncol. 2025 Jan 8;51:100915. doi: 10.1016/j.ctro.2025.100915 (PMC11783056; doi:10.1016/j.ctro.2025.100915)

Supplement Figure 1


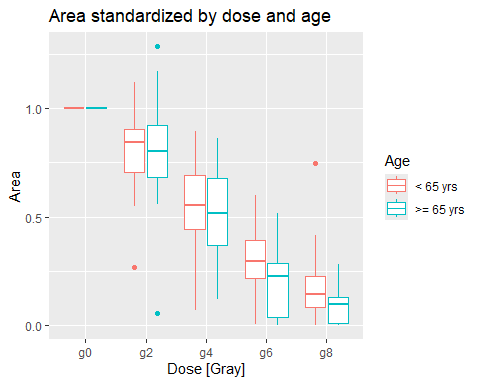

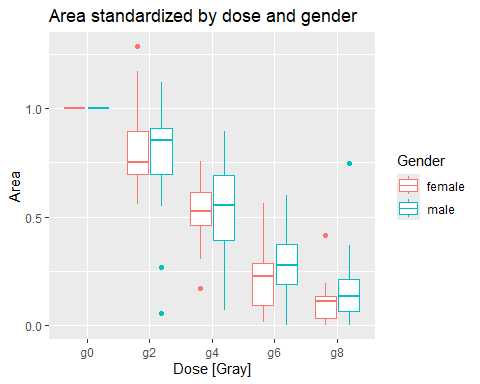


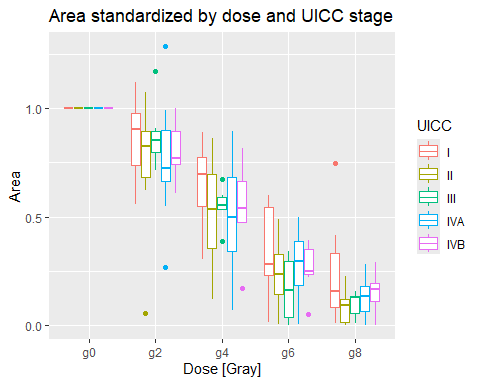


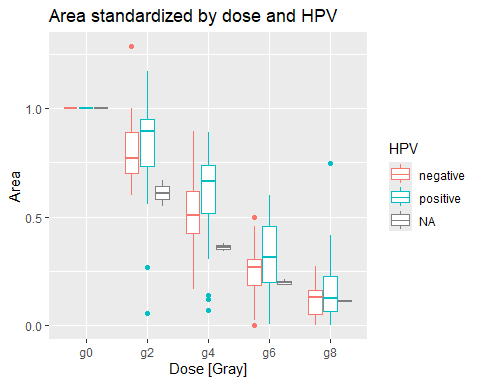

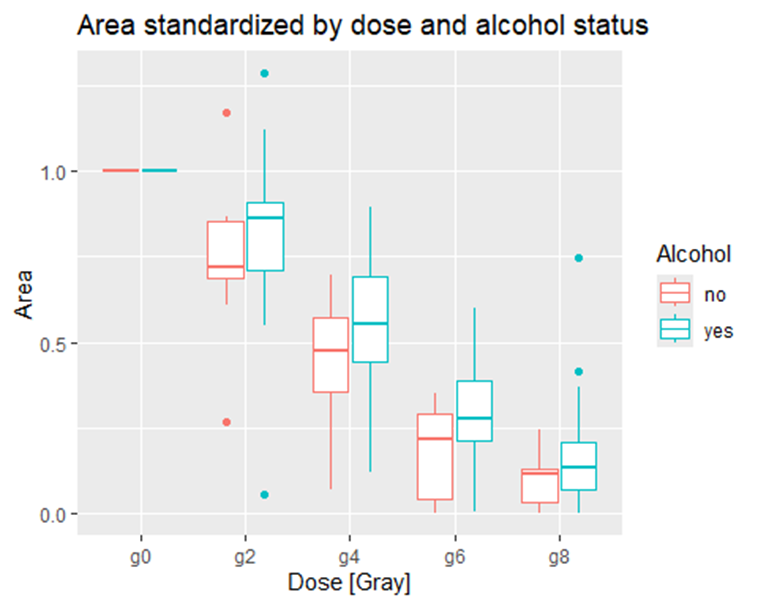

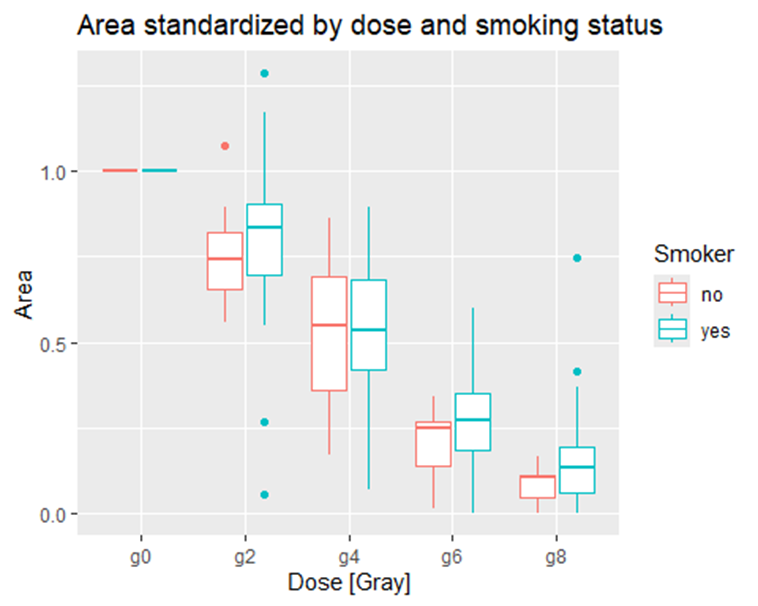


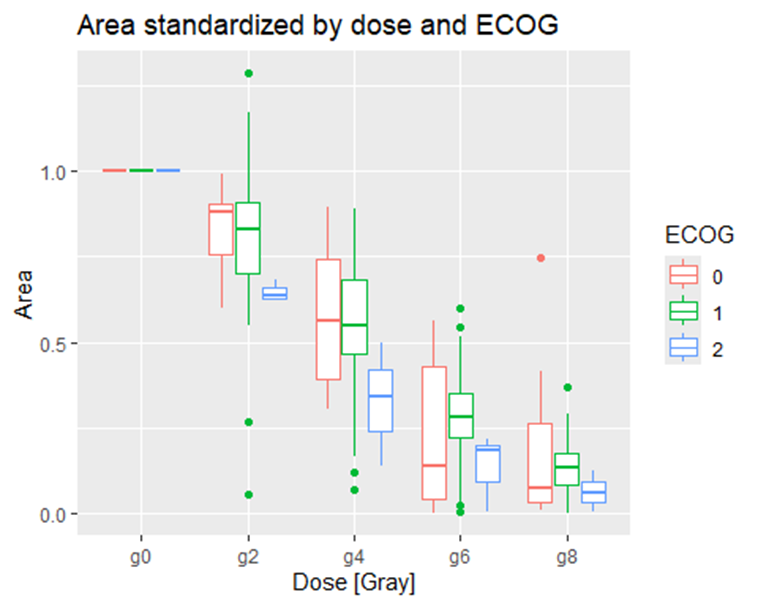

Supplement: Supplementary Data 1 [file mmc1.docx]
